# Maxims of Military Surgery

**Published:** 1861-05

**Authors:** 


ART. IV.—*Maximen der Kriegsheilkunst.* Von Dr. L. STROMEYER, Kœnigl. Hannov. General-stabartze, etc. etc. etc.

*Maxims of Military Surgery.* By Dr. L. STROMEYER, Physician of the General Staff of the Royal Hanoverian Army; Commander in the Second Class of the Royal Hanoverian Guelphic Order; Foreign Honorary Member of the Medico-Chirurgical Society of London, and the Chirurgical Society of Paris; Knight of the Royal Prussian Red Eagle, 4th Class, and of the Order of the Royal Saxon Civil Service; Member of the Imperial Russian Academy of Medicine, the Royal Belgian Academy, etc. etc. Hanover: Hahn's Royal Bookstore, 1858. 2 vols. 12mo., pp. 773, and octavo Supplement, 1861, pp. 150.

IT is indeed sad that there should exist motive for fearing a peculiar appropriateness to the study of military surgery in these our hitherto happy United States. Such, however, would seem to be the unfortunate fact. Let us hope that this will speedily cease to be the case. The nation will never be without an army; and there can be no doubt of the necessity, at all times, of cultivating this important branch of medical science.

Dr. Stromeyer had a favorable opportunity of studying large masses of gunshot wounds in Friburg, during the unhappy war of 1848; and was afterward, from 1849 to 1851, physician to the general staff of the army of Schleswig-Holstein. After the termination of the war, he tells us that his first intention was to incorporate the results of his military experience in a volume of his Treatise on Surgery; but that the magnitude and importance of the work and the space it occupied in his mind, compelled him to give it the separate publication which it so obviously requires.

In his introduction, Dr. Stromeyer urges the necessity, in addition to a university course, of an elaborate study of the best practical authors. The individual observations of the military practitioner ought to tend to qualify or confirm some principle; and this can only be done so as to improve the art, when adequate care is taken to become already familiar with the works of those who have gained a high reputation by their previous publications on this subject. He ascribes a just valuation to the labors and experience of Larrey, Guthrie, Hennen, and Langenbeck; and earnestly wishes that those to whom the instruction of medical youth is intrusted would, by every means in their power, promote the study of these authors. It would, he believes, be a valuable service to humanity and science, were some one to compile a fair abstract of all that has been done in this branch of our profession since the invention of gunpowder. In his reflections on this subject, the conscientious author makes some remarks upon certain discussions in the Paris school, at which we were tempted to smile. He prefers the inductions of the above experienced and painstaking men to "the fluctuating Paris manufacture which the discussions (on gunshot wounds) in the Academy of Medicine afford; from which anybody may pick out what he pleases; which, however, shall represent no principle, but merely communicate a mass of partly contradictory, individual views." It is not improbable that, in countries where the government recognizes the value of the medical profession, protects its practitioners, and grants emoluments and honors to those who are eminently useful to mankind, the necessity of mutual support, by deference to the opinions and feelings of a competitor, may be less keenly felt than among the Anglo-Saxons of perhaps either side of the water. In a land where acts of incorporation are not given to homœopathians, eclectics in a sense very different from the historic, and female colleges, it may be so. Certain it is, that the writings of Dr. Stromeyer are eminently characterized by a truly German love of truth, and by a desire for public utility. The position of the military surgeon renders it scarcely possible to extend the science of war-surgery, unless he is placed in a hospital station. During a long peace, too, the impressions on the memory are weakened, and partially superseded by more recent occurrences. Dr. Stromeyer does ample justice to the ardent aspirations, so often cruelly disappointed, with which many young men enter the medical service of armies; but it is the duty of a philosopher and a man of business to contemplate things as they are, and to labor after, not what is best in the abstract, but what is obligatory and most useful in the existing state of things. The character of the experience gained, too, varies excessively; and examples are seen of army surgeons who have neglected to practice attendance on the wounded, and have first become familiar with bandaging on an actual field of battle.

In regard to their qualifications for this service, physicians fall into the following classes:—

1. Such as, by the variety of their talents, by their character, their experience in service, and their vigorous health, are competent to every duty.

2. Such as, by their vigorous health, by peculiar adroitness in their intercourse with officers and men, are peculiarly qualified for service among marching troops.

3. Such as are peculiarly qualified for the treatment of in and out patients of hospitals.

4. Such as, from possessing the character of experienced surgeons and operators, are peculiarly qualified for the service of ambulances.

5. Those, whom a peculiar fitness for and inclination to administration, point out for the management of muster-rolls and accounts. A well-organized army develops these men; but in a newly-raised body they belong to the exceptions.

6. Those, in fine, who should be got rid of as speedily as possible, as they do no particular honor to their station. On account of such individuals as these, where sudden and large augmentations are made in the numbers of the military medical body, the appointments may be made provisionally.

These qualifications, says Dr. Stromeyer, cannot remain long concealed from an observing chief; and form, in his opinion, a much better basis for appointments in war than the over-estimated rolls of seniority. The latter consideration, when compared with fitness, should, in our author's opinion, be disregarded.

The author believes that in the north of Germany the service has been improved by the decay of the special schools for military medicine, and the assumption of competent men from the universities.

These considerations of the qualifications of surgeons, though an important special subject, are included by our author in his introduction. To do justice to his learned and experienced inquiries in a review is impossible: they are strongly characterized by terseness. We will, however, sketch the arrangement of his work.

The appointment of army physicians,—(we dislike the servility of employing the French word personnel,)—that of apothecaries, the equipment of the health establishment, the hospitals, the ambulances, the general regulations and instructions, and the mode of drawing up reports,—these occupy forty-five pages, constituting the already-mentioned introduction. If we could use the freedom, we would say, that it is a very important section, but does not introduce anything.

The first section ensuing is on wounds in general, and almost exclusively on gunshot wounds; the next, on the general course of the symptoms of them; then one on the treatment of wounds on the field of battle; then a special article, of moderate length, on bandages and other dressings. Twenty pages are enough for the treatment in hospitals.

The second volume is on particular injuries. First, on an account of injuries of the head from gunshot wounds; then an elaborate analysis of the symptoms which may ensue from them; then follow with rapidity injuries of the face and neck. Thirty-three pages are given to the thorax; and then follow, in a somewhat abridged form, injuries of the abdomen, pelvis, spine, and upper and lower limbs; three pages on artificial limbs; and then a table of injuries of bones of the extremities; and one, of the number of persons invalided for wounds.

The Supplement contains the principal additions made in the second edition of 1861, here furnished along with the first. Its articles relate to the construction and management of barracks, the treatment of the principal diseases of soldiers, an extremely neat series of wood-cuts of morbid specimens, and a translation of the British regulations to reform the army medical department.

As our space is limited, we shall be obliged to make selections; and we fear doing so without adequate means of discrimination; but, as the Supplement possesses the advantage of bearing a more recent date—that of the present year, 1861—and as the labors of Dr. Stromeyer are never deprived of their just circulation, but always enter into the science of the time, we shall give the preference to this latter volume.

*Regulation of barracks.*—No barracks, however roomy, can dispense with ventilation; they will inevitably become unhealthy if deprived of it; and the most important of the injurious results in the Schleswig-Holstein war were the formidable pestilences of typhus fever and granular ophthalmia. Yet ventilation, at last, can only in part make up for the want of sufficient breathing space within the chambers; and rooms positively overcrowded will be injurious to health, although with the most perfect arrangements of this kind. Even under the open sky, the assemblage of vast masses of men is found to generate disease.

In the best barracks of the Schleswig-Holstein infantry, 700 cubic feet per man were provided, including the double purpose of residence and sleep; and it was not difficult to obtain 800, on the occasional appearance of granular ophthalmia. In the English regulations of 1859, 600 are established by government. It is of great importance to have the sleeping and the sitting rooms separate, so as to subject the bedding to currents of air. A high, dry, well-drained situation is required, without hollows that can contain water, without mud, and protected from the drainage of neighboring grounds. Abundance of good drinking water must be accessible.

The size of the grounds will depend on the situation; but it is the more important as impediment to ventilation exists. A southern exposure, admitting of a majority of the windows in that side, is the best; next to this, a southeastern. It will be recollected that the ocean lies on a different side from that which it occupies in the United States. Taste should never be allowed to interfere with this. The barracks should include no hollow square, or much-projecting wings—never to exceed a projection of 25 feet; 500 feet should be allowed for the sleeping-rooms, and 300 for the day-rooms; with large communicating openings near the ceiling, closed with Venetian blinds, to be left open in summer, after airing the day-rooms. All the rooms should be on the southerly side, with the corridor on the north. The windows of the corridor must be opposite the doors of the rooms, which should be folding-doors. The utmost deviation from these rules which can be permitted is the construction of two short wings to contain small chambers. For economy, the chambers should be made somewhat deep from north to south, and proportionably narrower in the other direction.

Old Philadelphia readers will be reminded by most of these instructions of the admirably-constructed Bush Hill Yellow Fever Hospital, now removed for the erection of dwelling-houses. The city owed this to the science and care of a commisssion of physicians; principally, we believe, to the labors of Dr. Samuel Powell Griffitts. As far as we are informed, we believe that no epidemic ever prevailed there, except occasionally a little intermittent fever at its western end.

The whole building should, for the sake of health, be made with deep cellars. This differs from some English and American authorities. If a perfect drainage be accessible, the washing-rooms should be placed there; if not, in the small projecting wings at the end, which should have solid floors, without cellars, and be themselves well drained. The bath-rooms should have hot water from the kitchen. The privies should be separate from the urinary funnels; and both remote from the dwelling-house. A stream of water should run through the bottom of the urinary conveniences. The apothecaries' shops must have good light, and a surface of at least 350 square feet.

Large buildings have recently been erected in Hanover, in accordance with these rules; and supplied with a parade ground of fifty acres.

*Hospitals for peace.*—Dr. Stromeyer frankly expresses the conviction, that the proper erection of these is the most difficult task of an architect. *It cannot be done without the advice of physicians;* and, as these are not specially instructed on the subject, and have rarely given it any particular attention, they are imperfectly qualified, and are obliged to instruct themselves as they go, to the great detriment of the service proposed.

It is not, certainly, the City of Philadelphia that has a right to upbraid another country than its own for inadequate investigation previously to building hospitals. The expensive erections which surround us, some of them costing enormous sums, have in some instances been produced in the manner which Professor Von Stromeyer considers impossible, without medical advice, or at least in defiance of it, and by the judgment of citizens engaged in other professions. In some instances, costly and splendid structures, now and for years without a patient, have been said, upon the highest authority, to have been built without inspecting elaborate reports which had occupied the time and industry of commissioners composed of medical men of standing.

Philadelphia, it is well to understand, however, is not without evidences of a proper influence exercised in the task of hospital building by those adequately qualified to give advice and superintendence. We have already mentioned Bush Hill. Dr. Rush's discourse on this subject, in his Sixteen Lectures, was written at the request of an active manager; and, at the time when that celebrated physician exercised the greatest influence on men's minds, served very extensively to diffuse a recollection of this important subject among the medical men of our country. The Lazaretto is well constructed; and was, we believe, made so with medical assistance. It was at a time when the terrors of the yellow pestilence were recent in men's memories, and a deep anxiety was generally felt for the mixed benefit of science and experience. The Naval Asylum, certainly a very fine and well-arranged establishment, enjoyed the services of an able medical staff. The military guard kept at Philadelphia has not yet, we believe, been thought large enough to require a considerable hospital building; but no one will question either the ability of the faculty, or their proper influence in this important duty.

Dr. Stromeyer is of opinion that the construction and management of hospitals should be regularly taught in universities by public courses, for the information of the young men in pursuit of medical honors; and that governments would then never experience any difficulty in selecting persons properly qualified to discharge these important duties.

In constructing military hospitals, part of the trouble is saved by the avoidance of the necessity of providing for both sexes. The idea has been relinquished, that it was desirable to assemble great numbers of patients with the same complaints in large wards; those more recently prepared not containing, in general, more than a dozen beds. This Dr. Stromeyer prefers, conceiving that the object of classification requires smaller rooms. In the General Hospital, at Hanover, there are rooms of three sizes; thirteen that contain sixteen beds each, sixteen for four beds, and eleven provided with only one or two a piece. It is difficult to estimate with confidence the relative proportions of mild cases and of the severe ones which any hospital is likely to receive; but by ascertaining the facts from similar institutions, it is not impossible to approximate to this result. The milder cases, for convenience, should be grouped in the larger rooms; but smaller ones are indispensable, from the necessity of separating the severe cases, and frequently of insulating them. The motives for this will sufficiently suggest themselves: silence; freedom from interruption; the necessity of peculiar cares, sometimes unpleasant to others; a peculiar state of the atmosphere, and of light; and lastly, the presence of those very ill or dying, with all the injurious effects upon the minds of those who are ill themselves, and often in a situation really, or in imagination, similar. Dr. Stromeyer hence finds it necessary to intersperse the large wards with a sufficient number of smaller rooms to answer these purposes. Some changes which have been made in our city, of smaller rooms into larger ones, and at considerable expense, will not seem to correspond with the conclusions of this eminently practical writer.

Hospitals are built two or three stories high from motives of economy. This Dr. Stromeyer defends; and directly opposes himself to the justly celebrated Miss Nightingale. His hospital, including a basement, is four stories high. His displeasure with this lady seems to us to be rather too vehement, and to have caught from her a little of that impassioned fervor for which he blames her. In his opinion, the rise of effluvia from one floor to those above it, is in no degree to be feared, if simply each different story is properly ventilated. The construction advocated by Miss Nightingale is, in his eyes, sufficiently objectionable from its nearly doubling the cost of erection. In addition to this, he conceives physicians called upon to oppose it, because, as he says, it excludes the use of smaller individual rooms for separating patients, as we have above described. According to him, this plan would compose the whole hospital of nothing but a series of buildings, each containing two wards, one above the other, and only communicating by open arcades. We have not the book at hand; but our recollections from the perusal of it have not left so strong an impression on our mind. We cannot see, amid Miss Nightingale's arcades, and her architecture so far too liberal in its expenditure, why there should be any difficulty in constructing the separate rooms required. This benevolent and distinguished individual has exerted herself, certainly with great warmth and energy, to procure more adequate accommodations for prolonging and saving the lives of the British soldiery; and it is difficult for us to think that she, or those whom she advises, would refuse to let them benefit by the experienced suggestions of Professor Stromeyer. If governments will not furnish the money, the objection is conclusive; but they certainly do, in some instances, squander a great deal that had been better spared. Dare we ask whether Dr. Stromeyer has forgotten two considerations which we shall cite? One is, that statistics have shown, as in the case of the hospitals of Paris toward the end of the reign of the first Napoleon, that the mortality of the second story of these establishments is greater, de facto, than that of the first. The tendency of the warm effluvia of an inhabited house to rise upward is a constant agency, perpetually in action; and the time allowed by any incompleteness, imperfection, forgetfulness, or delay, is never wasted by that unfailing power of nature, the difference in specific gravity. If the ventilation of all the stories be absolutely perfect, and there be no staircase or other means of communication, the rise of effluvia within the house might be prevented. Even then it will take place to a sensible extent up the outside walls of the building. Professor Stromeyer shows how a current can be sent through lower openings on the north and cooler side, and more elevated passages in the southern and heated surface. But the operation of these processes is not perfect; nor can any attendance to close doors be safely calculated on as such. *Quis custodiet custodes?* Why place human subjects in a situation where the evil is certain and unremitting, and the protection liable to imperfections and exceptions?

The other objection which we beg leave to state, is that the climates of which the learned professor and the benevolent lady write, are essentially so different. They both censure the practical evils which they respectively saw; and it appears to us quite reasonable to suppose that the rapid chemical changes of the vicinity of Constantinople, the habitual exposure of large masses of putrid matter, and the neglect of drainage, required a far greater volume of fresh air to neutralize their destructive effects than the corresponding circumstances in such a country as Hanover.

Air-heating furnaces, in the opinion of Professor Stromeyer, are only suited for sick chambers. Ventilating draught tubes, terminating in chimneys, or above the top of the roof, are of much service. They can produce a sensible freshness in the air of the smallest chambers, and readily supply abundant air to patients with diseases of the eyes without making the room brighter.

Several other mechanical details, of real value, are given; but we are obliged to omit them for want of space.

The results of careful experience in the general hospital are given to us in a medical report, which fills sixty-nine pages of the volume; and from which we extract the following matter. The observations embrace the last ten years. Nearly 10,000 troops were posted in the capital during that time; a few cases of the worse class were sent there from other military points, and some civil cases admitted. The number of deaths among the troops averaged but $5\frac{2}{10}$ in the thousand; while that of male individuals of the same age in the whole kingdom amounted to $9\frac{5}{10}$ in the same number, and, in the capital, to 10; thus proving that the preservation of life was better among the troops than among the citizens. The situation of the capital city was not thought one of the most desirable in respect to health. The cases in the hospital from 1854 to 1859, inclusive, and in the last four months of 1853, were so selected or intercepted as to amount to the exact number of 10,000. Of the 242 cases of inflammation of the lungs, no death is acknowledged; of 60 of consumption, 30, or one-half; of typhus, 18 in 225, equal to 7·2 per cent. The following are set down without a single death: catarrh of lungs, stomach, and bladder, 1120; abscess, 332; pharyngitis, 566; undeveloped diseases, 1819; measles, 45; syphilis, 371; intermittent fever, 771. We make the total of these last 5024.

The professor sums up the general impressions which his observations have left on his mind in the following dogmas or propositions, which he submits with great modesty to criticism. The degree of correspondence which may exist between this Ideal and the Habitual, in the practice under his superintendence, he refers to his accounts of particular diseases. These form a most valuable mass, the thorough study of which is desirable to any man; but to which our limits, were it not for our doubtful competency, render it impossible to do justice. As, however, they bear the more recent date, 1861, partial abstracts from them will probably fill the greater part of our remaining pages.

1. The healing art is founded upon the natural history of disease. A good therapeutic method must follow the physiological processes which are disordered in the patient. This includes injuries which are the subject of surgery, and also poisoning.

2. There are no specific remedies. All that appear such should be explained physiologically.

3. Regulation of the mode of life of the patient is the most important problem which is laid before the physician, as it relates to the action of all the stimuli which support and modify life. This is equivalent to diet, in the original Hippocratic sense. (*Foes, sub verbo.*)

4. Medicine should never be given till the mode of life is previously regulated. To practice medicine in foul air, or during the use of improper food, is like making chemical investigations with impure reagents.

5. Drugs should be given when they are necessary or *euphemistic*, and when they are indicated. By the word which we have expressed in italics, we presume that the author means in good repute.

6. A good medicine must be one which will never add to any of the detriments to which the patient is liable to suffer in the course of the disease. Such a remedy should be considered faulty or imperfect.

7. The natural history of the disease includes the ground-work of its existence; and this leads to the most noble problem submitted to physicians—the prevention of disease.

Military physicians are more frequently than others in a position to render services of this kind.

"These maxims," says our author, "characterize the true physician; and the reverse of them, the quack. Let them be to us words of counsel, beloved as the beautiful constellations that guide the ship through the ocean."

The first article of Surgeon-General Stromeyer's pathological report is a note on acute exanthemata. Of these, in his one thousand cases, there are forty-six of scarlatina, forty-five of measles, and four of windpock. We suppose that this last affection would here be called a mild varioloid. It is not in our dictionaries, but in Van Swieten, v. 10. There was no small-pox; "an immunity," says our author, "beyond doubt to be ascribed to the abundant revaccination of the recruits;" a fact worth copying in our newspapers, to nullify the clamor sometimes raised by unprincipled or ignorant persons. The small-pox constantly prevailed in the city; and in the army, out of one thousand revaccinations, thirty-eight, on an average, were successful. The scarlatina cases were distributed throughout six years; of the measle cases forty-one in forty-five occurred in one year. The patients affected with measles or scarlatina were not removed to the insulated house for contagious cases, but merely placed in separate chambers. To the effects of good ventilation (added to general good management) Dr. Stromeyer is inclined to ascribe the fact that no contagious case, no morbid sequelæ, and no death from these cases occurred in all that time. The pulse, in the scarlatina cases, never exceeded eighty in the minute!

*Rheumatism.*—It is difficult justly to divide the cases into acute febrile and non-febrile; the state of the weather having so much influence on this point, that observation continued for some time is necessary for the discrimination. They fall more naturally into the three following classes:—

1. General acute rheumatism of joints.
2. Local rheumatism of joints.
3. Painful rheumatoid condition, without deposits capable of being pointed out.

General acute rheumatism of joints declares itself immediately after exposure. After a severe chill on guard, or after a forced march, fever and swelling of the joints come on so rapidly that the subject must be sent immediately to the hospital. The fever is absent in but few cases. Generally the ankles and knees are affected: one knee more severely than the other. The inflammatory process seldom subsides rapidly; but the disease is further developed. The finger-joints, wrists, and elbows are soon seized; but prior to this, Dr. Stromeyer has found, as the rule, that infiltration has taken place in the lungs; the physical signs of which, in general, rise higher on one side than the other, and in some instances as high as the shoulder-blades; the indication being obtained by percussion. A fine mucous rattle is generally present; but not in every instance; rarely cough or dyspnœa. The affection of the heart does not accompany these; but follows the extension of the disease from the arms. It cannot, therefore, arise from the prone position of the patient. Undoubtedly there are exceptions to this order of succession; and he has even seen cases of endocarditis and pericarditis in which no signs of the rheumatic process had made their appearance.

When cases are daily examined with care, the first evidence of diseased heart is a change of the rhythm of the organ's motions from a prolongation of the first tone. Here the affection often stops; but if it be further developed, the endocardial rustling is heard. In patients treated early and promptly, as Stromeyer's generally are, pericarditis rarely ensues; and not till there is previously well-marked endocarditis. In the majority of cases, one joint is more severely affected than the others throughout. Leeches give much more relief than bleeding. It consequently almost appears as if the inflammatory status of a part operated to derange the composition of the blood; and, probably by increasing the development of fibrin, gave room for the development of the rheumatic process. It is twenty years since our author proclaimed that the augmentation of fibrin in the blood, during the phlegmasiæ, was not the cause, but the consequence, of the inflammatory process, and stood in connection with the impediment to the circulation in the inflamed part, and with the increased development of heat. It is a circumstance instructive in this respect, that the augmentation of fibrin in the blood is often wanting in inflammatory affections of organs of small size, as these cannot essentially change the whole mass of the blood.

Affections of the brain never appeared among Dr. Stromeyer's cases. But a single death of a rheumatic patient occurred in the whole number of five hundred and thirty-two instances of rheumatism; and this, before his service commenced. His accumulations of facts, therefore, on this point terminated with observing the phenomena of endocarditis by the sick-bed, and generally with friction sounds and effusion, without the unhappy advantages of dissection.

*Treatment.*—In his experience the cool treatment of rheumatism proved itself the most important principle of cure. Patients became worse, or were delayed in their recovery, in exact proportion to their proximity to the stove. He was, by his success, gradually led to abolish the customary load of bedclothes; and, in summer, even to cover his patients with nothing but a linen sheet, unless, indeed, they asked for a blanket. This procedure had the happiest influence. Until he adopted it, he had frequently the regret to see patients, who were admitted for a simple affection of one joint, become gradually involved, under every medication, in a general rheumatism accompanied by fever. This afterward entirely ceased to be the case; and the removal of rheumatic subjects to a cool room became established in the hospital as a useful resource. In the same manner cases were more successfully treated in winter.

Dr. Stromeyer has entirely relinquished general bleeding; not from the production of an inconvenient debility by that means, but from observing its little utility. Leeches were found more useful than cups, from the long continuance of the bleeding they produce. Still, he is far from wishing to restrict the use of cups. To keep up the oozing of blood from the leech-bites, sponges were not so successful as wet compresses. The former insinuated their fibres into the bites and produced little abscesses, accompanied with considerable increase of the existing pain. "It was easy to be deceived as to the cause of this increase; and equally so to make it suddenly cease, when, by the application of warm cataplasms, the little abscesses were made to empty themselves." We would ask, how long the sponges were kept applied? and, if we could make so free with this eminent teacher and administrator, whether he had forgotten that simple cataplasms would relieve rheumatic pain?

Leeches should be applied to the region of the heart as soon as the physical signs of endocarditis appear, without waiting for the occurrence of pain, as some high authorities have advised. Pain in that region indicates too advanced a stage of pericarditis to be waited for.

Dr. Stromeyer believes opium the only intrinsically effective internal medicine for rheumatism; and conceives that, in regard to the employment of any other drug in the case, the question really is merely how to prepare, or keep prepared, the patient for the use of this crowning remedy. This is to be performed by diminishing the distention of the blood-vessels, and by attention to the bowels. He reduces the force of the pulse with nitrate and bicarbonate of soda, and, if the tongue be then moist and clean, immediately administers opium.

Against the derangements of the stomach, by which the effect of morphia is interfered with, he has relinquished altogether the use of antimoniated tartar. If a deposit begin to form on the tongue, the nitrate of soda is laid aside, phosphoric acid given, and the use of the morphia intermitted, until the tongue again becomes clean and moist. To avoid disappointing patients of their beloved "sleeping powder," they are furnished with pulvis sacchari.

Dr. Stromeyer has also given morphia in endocarditis and pericarditis, according to the method of Dr. Billing, of London, but not in equally large doses; not deeming it necessary, and being "perhaps" led by the preceding cautions. A quarter of a grain once a day is generally sufficient; sometimes this being given twice in the same period, but very rarely more frequently. Digitalis is greatly inferior, does not relieve the pain, and disturbs the stomach. Castor-oil is used to regulate the bowels; and the diet adjusted according to the pulse. By the cool treatment the profuse sweats are avoided. These he hopes "none will, at the present day, consider critical." Strong sudorifics should be entirely discarded, more especially during acute affections of the heart; a state of things which it is not easy to alleviate with leeches and morphia. Calomel was given for this object, in the dose of one grain every two hours; but seldom to a larger amount than twelve such doses.

By this treatment all his patients recovered; the heart symptoms ceasing, except a few cases, in which a slight systolic rustle could be discovered, when they were discharged.

*Local rheumatism of joints.*—These cases almost always have the form of inflammation of the synovial membrane, or dropsy of the joint; unless they are relapses or second or later attacks in the same joint. They have generally the appearance of injury of joints from gymnastic exercises. They occur commonly in the knee. Sometimes the inflammation is very severe, and requires repeated local bleedings, the application of ice, and the use of mercury to a considerable extent. Against the more chronic form the principal resources were fomentations with vinegar and water, and sometimes blisters. The use of morphia for pain and to procure sleep should not be omitted. In all rheumatic affections of joints great care is called for to obtain and preserve proper attitudes for the diseased member.

*The condition of "being rheumatic."*—The pains and impediments to motion, which appear under the form of muscular rheumatism of single joints, or of the thorax, or in that of lumbago or sciatica, were more generally reflex phenomena from the large bowels, which were at the time laboring under a catarrhal affection, and loaded with masses of hardened fæces. (We should probably call it colic from cold.) Hard masses were felt on palpation of the surface of the abdomen, and the patients were relieved when these were evacuated. This, however, was not always the case; and the exceptions were mostly treated with a little morphia and by warm fomentations with cloths wet with decoction of flax-seed; cups and blisters being seldom needed. Sometimes warm baths were used; and, in a few obstinate cases, iodide of potassium, oil of turpentine, or corrosive sublimate.

The good effects of colchicum appeared to him to be owing partly to its combination with opium, partly to its purgative operation. When this last depended on the colchicum itself, the operation amounted, in some cases, to a poisonous one. He therefore considered the plant an unsafe medicine.

Rheumatism of the muscles of the neck, the simplest form of that rheumatism which arises from the action of the nerves of the skin, never appeared among his soldiers.

With this we are compelled by our limits to close the very partial analysis which we commenced on the valuable and eminently practical work of this distinguished physician. It would be a grievous loss to the Anglo-American profession were it not to be translated. It would give us great pleasure and render a substantial service to our readers to carry our review through the remainder both of the present volume and of the two earlier ones. Whether this will be done or not, will depend upon the convenience of this journal.

